# Supplementary material for: Bayesian Phylodynamic Analysis Reveals the Dispersal Patterns of African Swine Fever Virus
Source: Viruses. 2022 Apr 25;14(5):889. doi: 10.3390/v14050889 (PMC9147906; doi:10.3390/v14050889)
Supplement: Supplementary file 1 [file viruses-14-00889-s001.zip › Supplementary File.pdf]

Supplementary File

1. The annotated files of ASFV genome.
2. The trimmed file of complete genome.
