# Supplementary figures and images for: Bayesian Phylodynamic Analysis Reveals the Dispersal Patterns of African Swine Fever Virus
Source: Viruses. 2022 Apr 25;14(5):889. doi: 10.3390/v14050889 (PMC9147906; doi:10.3390/v14050889)

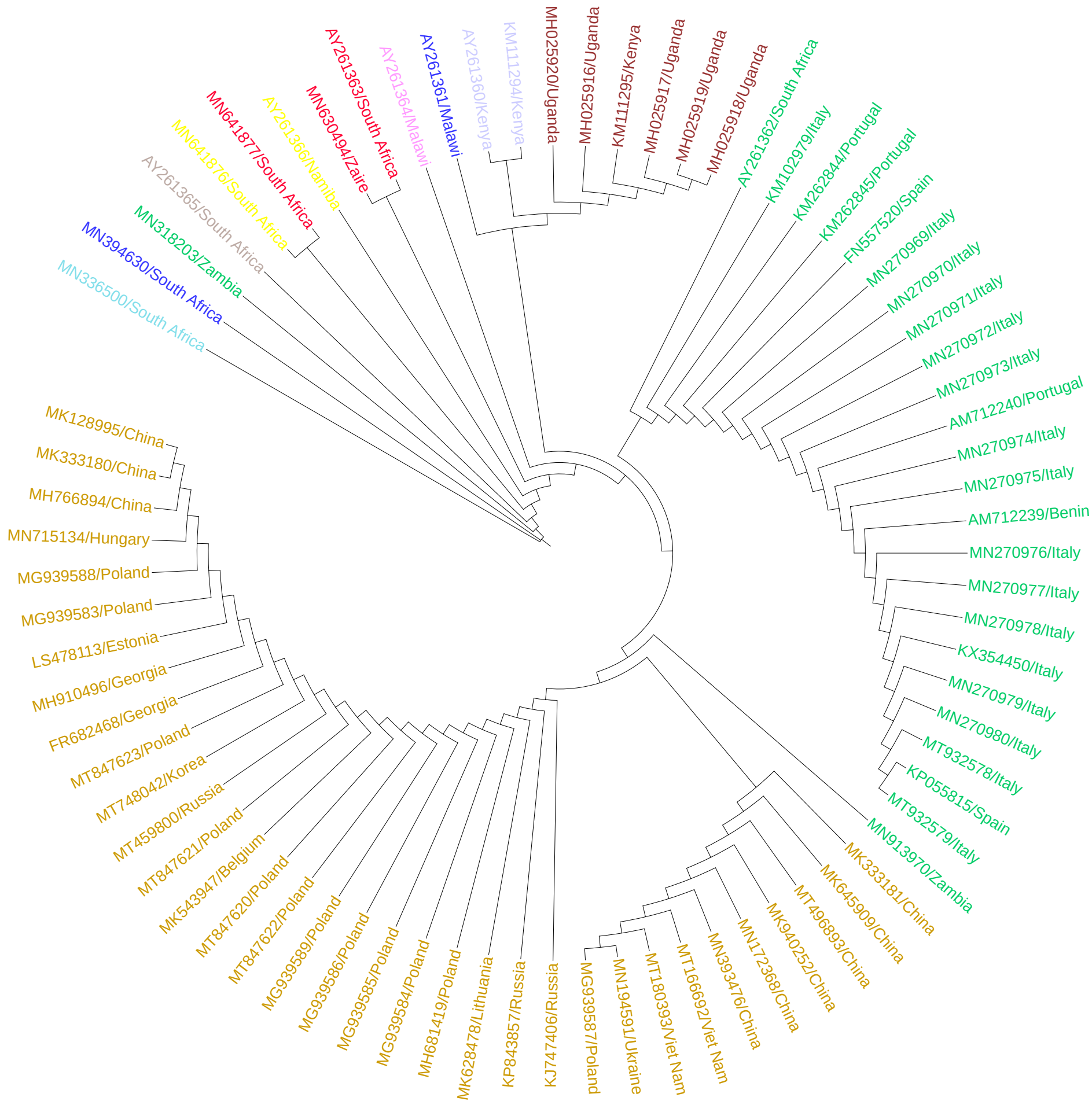

Supplement: Supplementary file 1 [file viruses-14-00889-s001.zip › Supplementary file S2/PhyML-E83L.pdf]

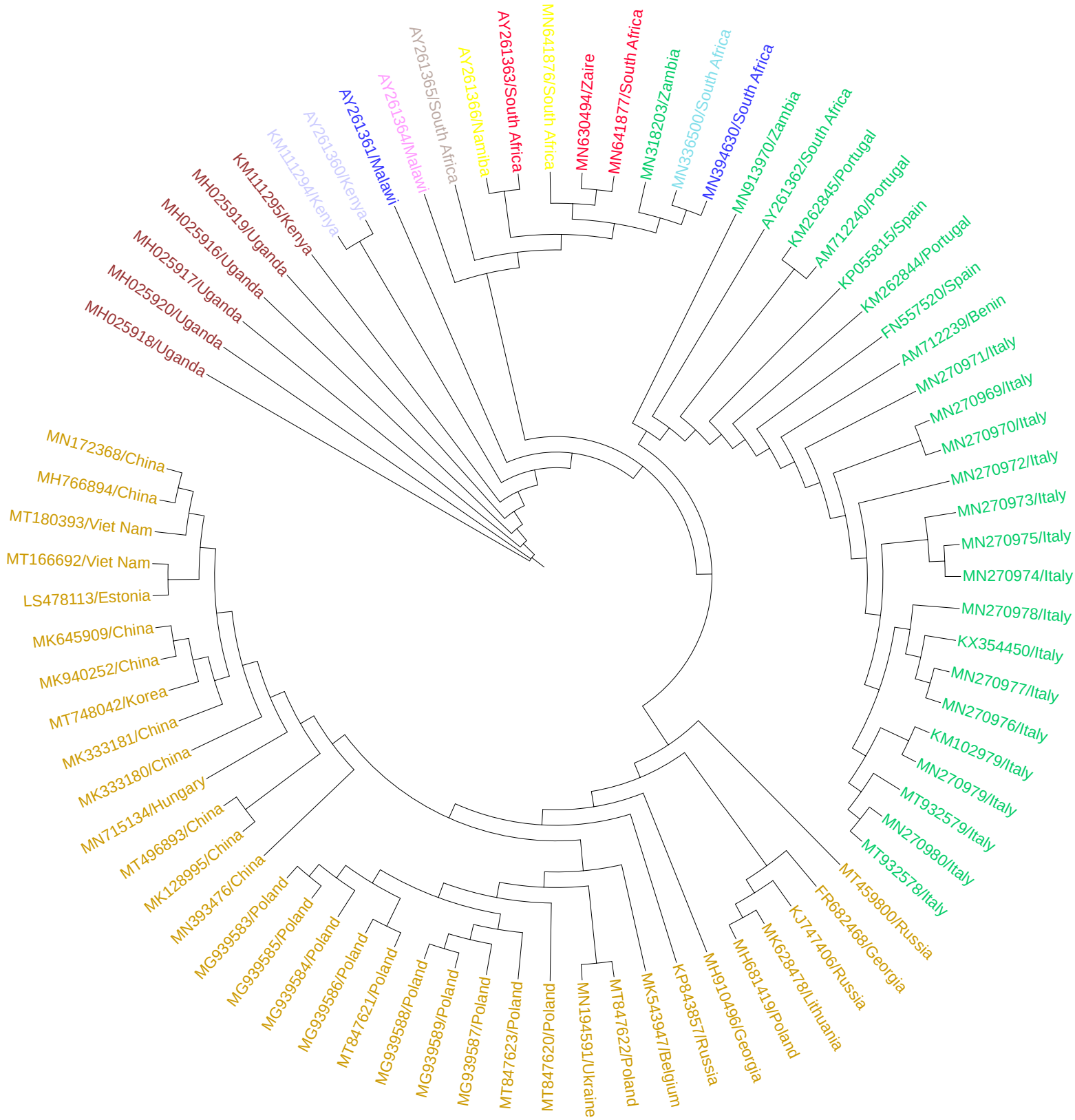

Supplement: Supplementary file 1 [file viruses-14-00889-s001.zip › Supplementary file S2/PhymL-genome.pdf]

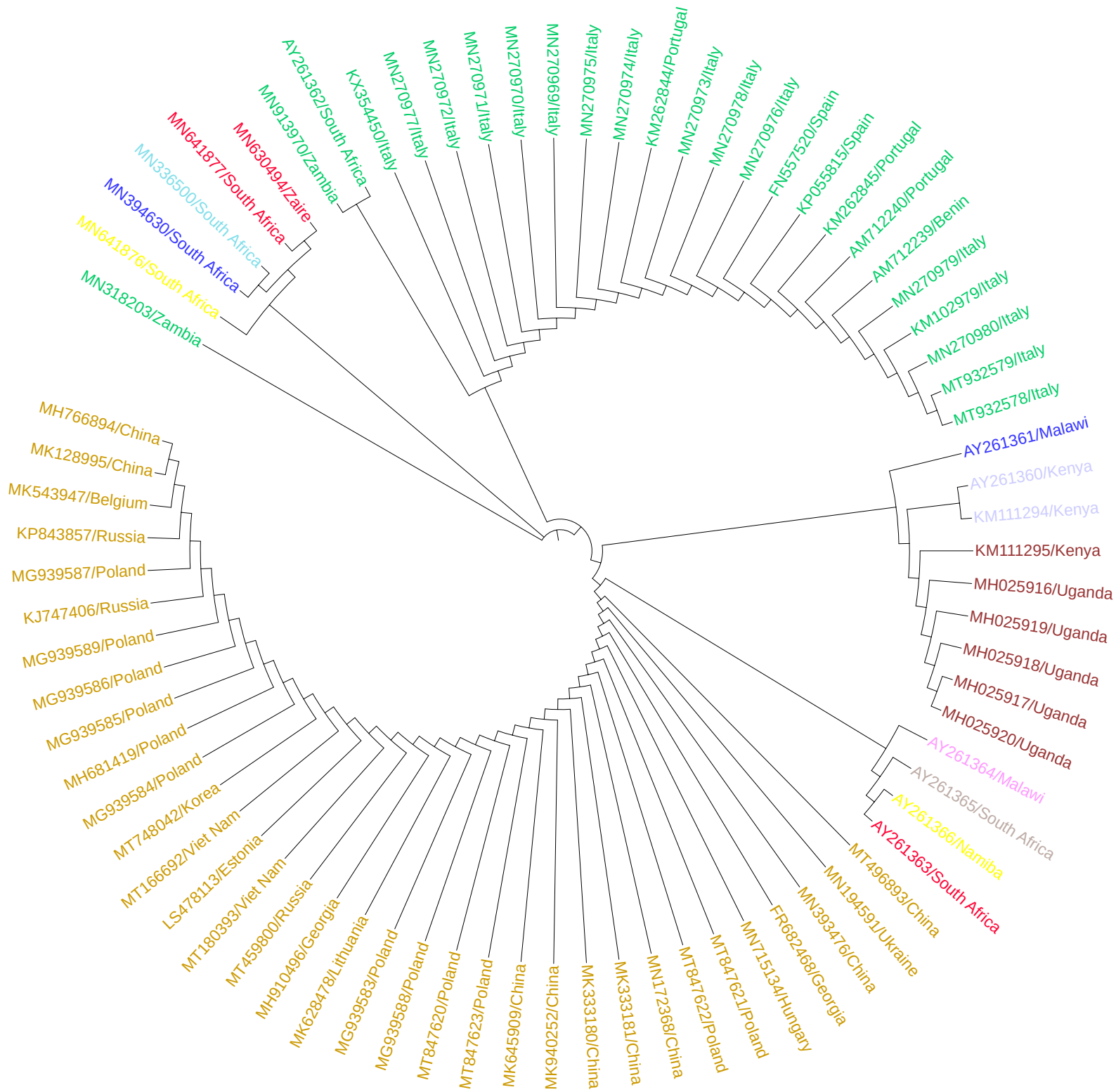

Supplement: Supplementary file 1 [file viruses-14-00889-s001.zip › Supplementary file S2/PyhML-B646L.pdf]
